# Evaluation of an automated protocol for efficient and reliable DNA extraction of dietary samples

**DOI:** 10.1002/ece3.3197

**Published:** 2017-07-07

**Authors:** Corinna Wallinger, Karin Staudacher, Daniela Sint, Bettina Thalinger, Johannes Oehm, Anita Juen, Michael Traugott

**Affiliations:** ^1^ Mountain Agriculture Research Unit Institute of Ecology University of Innsbruck Innsbruck Austria

**Keywords:** BioSprint, cetyltrimethylammonium bromide, DNA isolation, molecular gut content analysis, molecular scatology, trophic interactions

## Abstract

Molecular techniques have become an important tool to empirically assess feeding interactions. The increased usage of next‐generation sequencing approaches has stressed the need of fast DNA extraction that does not compromise DNA quality. Dietary samples here pose a particular challenge, as these demand high‐quality DNA extraction procedures for obtaining the minute quantities of short‐fragmented food DNA. Automatic high‐throughput procedures significantly decrease time and costs and allow for standardization of extracting total DNA. However, these approaches have not yet been evaluated for dietary samples. We tested the efficiency of an automatic DNA extraction platform and a traditional CTAB protocol, employing a variety of dietary samples including invertebrate whole‐body extracts as well as invertebrate and vertebrate gut content samples and feces. Extraction efficacy was quantified using the proportions of successful PCR amplifications of both total and prey DNA, and cost was estimated in terms of time and material expense. For extraction of total DNA, the automated platform performed better for both invertebrate and vertebrate samples. This was also true for prey detection in vertebrate samples. For the dietary analysis in invertebrates, there is still room for improvement when using the high‐throughput system for optimal DNA yields. Overall, the automated DNA extraction system turned out as a promising alternative to labor‐intensive, low‐throughput manual extraction methods such as CTAB. It is opening up the opportunity for an extensive use of this cost‐efficient and innovative methodology at low contamination risk also in trophic ecology.

## 1 INTRODUCTION

The rates and pathways of nutrient cycling through ecosystems depend on trophic interactions, and consumers play a key role in storing, recycling, and redistributing nutrients in any given ecosystem (Torrance & La Pierre, 2015). However, the variety of feeding relationships is often as complex as the diversity of organisms that are usually involved in ecosystem processes, including vertebrates, invertebrates, and plants. The theoretical framework of food web ecology is lacking of empirical field data that parameterizes trophic interactions (Albaina et al., 2010; Allesina et al., 2015; Ulanowicz, Hold, & Barfield, 2014). Accordingly, comprehensive investigations measuring the full trophic pathways are required (Bell et al., 2008), even though assessing the feeding interactions in complex trophic networks is challenging.

Molecular techniques provide an effective means of assessing trophic relationships, particularly in systems where these are difficult to observe with conventional nonmolecular methods (Pompanon et al., 2012; Symondson & Harwood, 2014; Traugott, Kamenova, Ruess, Seeber, & Plantegenest, 2013). A major advantage of using DNA for gut content analysis is that it allows precise identification of species‐specific predator–prey relationships. Moreover, apart from screening high numbers of consumers simultaneously, analyzing concomitant predation on multiple prey species is possible by either using diagnostic multiplex PCR (King, Read, Traugott, & Symondson, 2008) or next‐generation sequencing (NGS)‐based approaches (Pompanon et al., 2012). Taken together, molecular identification of trophic interactions has shed light into complex food webs (Davey et al., 2011; Eitzinger, Micic, Körner, Traugott, & Scheu, 2013; Hrček, Miller, Quicke, & Smith, 2011; Joly et al., 2014; Staudacher, Jonsson, & Traugott, 2016). Examining these feeding networks with molecular methods usually entails processing large numbers of samples: Raso et al. (2014), for example, tested more than 2,500 invertebrate predator samples for extra‐ and intraguild prey, and Gariepy, Kuhlmann, Gillott, and Erlandson (2008) screened DNA extracts of 26,000 field‐collected mirid host samples for the occurence of parasitoid DNA. The use of low sample numbers entails the risk of only capturing a smaller proportion of the actually consumed prey species (Burgar et al., 2014) and/or missing statistically significant differences in the trophic variables examined. Accordingly, high sample numbers, which are representative for the trophic interactions studied, need to be analyzed molecularly.

Here, DNA extraction often represents a bottleneck as, depending on the protocol used, it can be tedious, time‐consuming, and expensive. Moreover, the presence of contaminating and potentially PCR inhibiting substances can impede many of the subsequent reactions and techniques (Berensmeier, 2006; Zarzoso‐Lacoste, Corse, & Vidal, 2013). Accordingly, efficient DNA extraction protocols are needed to generate high‐quality DNA from various types of complex dietary samples that often only contain trace amounts of food DNA (King et al., 2008; Pompanon et al., 2012). Among the conventional extraction methods, a range of approaches is known for DNA isolation in the fluid phase. They involve lysis by a detergent or chaotropic substance (possibly in the presence of protein‐degrading enzymes), followed by several processing steps applying organic solvents such as phenol and/or chloroform or ethanol, which in general are toxic and require special and expensive disposal. Alternative separation techniques are employing solid‐phase systems, where sorption processes are based on silica under chaotropic conditions, ionic exchanges, as well as affinity and size exclusion mechanisms (for a review, see Berensmeier, 2006). These classical DNA extraction methods are not only laborsome and time‐consuming, but the relatively large number of steps involved increases the risk of DNA degradation, sample loss, or DNA cross‐contamination, especially when high numbers of samples need to be processed simultaneously. During the last few years, paramagnetic beads have been increasingly employed for DNA isolation, representing a relatively easy and inexpensive technology, subjecting samples to very little mechanical stress (Berensmeier, 2006; Suomalainen, Suomalainen, Puro, Kytöniemi, & Lamberg, 2010; Vidergar, Toplak, & Kuntner, 2014). They are particularly suitable for automated platforms such as the BioSprint^®^ 96 Extraction Robotic Platform (Qiagen, Hilden, Germany), the KingFisher^®^ 96, and MagMAX^™^ Express Magnetic Particle Processors (Thermo Fisher Scientific Inc., Waltham, MA, USA), respectively. Unlike classical DNA extraction methods, where reagents are moved into and out of a single well to perform the different steps of a DNA isolation procedure, permanent magnetic rods are used collecting paramagnetic beads to which the DNA is bound and released into 96‐well plates (Fang et al., 2007). Automated extractions obtain purified DNA in sufficient quality and purity and proved to be consistent and reproducible (Loeffler, Schmidt, Hebart, & Einsele, 2004). Centrifugation steps are avoided and sample handling steps are reduced, and thus, the risk of cross‐contamination is lowered. Compared to classical DNA extraction methods, the magnetic separation of DNA has several advantages: The simplified procedures employing a robotic workstation for magnetic particle handling entail increased worker safety together with reduced sample processing time leading to increased laboratory efficiency (Boyd, 2002). The method produces good yields of high‐purity DNA appropriate for a variety of downstream applications (Fang et al., 2007; Tan & Yiap, 2009; Wochner, Birgit Cech, Menger, Erdmann, & Glökler, 2007) and hence might be especially suited for large‐scale studies (Berensmeier, 2006).

In dietary studies, so far, mainly classical DNA extraction methods, such as cetyltrimethylammonium bromide (CTAB)‐ or silica‐column‐based approaches, have been employed and compared for efficiency in retrieving food DNA (e.g., Oehm, Juen, Nagiller, Neuhauser, & Traugott, 2011; Simonelli et al., 2009; Zarzoso‐Lacoste et al., 2013). This work has quite recently been accompanied by an increasing number of investigations on trophic interactions using paramagnetic separation techniques in combination with automated DNA extraction systems (Jarman et al., 2013; Oehm, Thalinger, Mayr, & Traugott, 2016; Roubinet, Straub, Jonsson, Staudacher, & Ekbom, 2015; Sint, Thurner, Kaufmann, & Traugott, 2015; Staudacher et al., 2016; Thalinger et al., 2016; Wallinger et al., 2015). Currently, several automated magnetic separators are commercially available (Berensmeier, 2006). Originally, these newly emerging automated methods have been conceived for extracting total DNA. An explicit testing of their suitability for dietary samples in comparison with classical DNA extraction methods is so far missing.

In this study, we tested a high‐throughput DNA extraction platform using paramagnetic beads for the detection of prey DNA in dietary samples from whole‐body invertebrate samples, vertebrate gut contents, and feces and provided an evaluation of its performance in comparison with a classical, well‐proven CTAB‐based DNA extraction protocol. Extraction efficacy was quantified using the proportions of successful PCR amplifications of both total and prey DNA, and cost was estimated in terms of time and material expense.

## 2 MATERIALS AND METHODS

Two different DNA extraction methods were compared, namely a CTAB‐based phenol–chloroform protocol (“CTAB samples”) and a silica‐based automated DNA extraction (“BioSprint samples”) on the BioSprint^®^ 96 extraction platform using the BioSprint^®^ 96 DNA Blood & Tissue Kit (Qiagen, Hilden, Germany). We selected different kinds of dietary samples to cover a broad variety of sample types, that is, gut content, faces, as well as whole‐body extracts, from different representatives of both invertebrates and vertebrates: 25 predatory arthropods (carabid beetles and spiders) from cereal fields, 52 ladybird beetles from a feeding experiment, 15 omnivorous carabid beetles of the genus *Amara* sp. from a glacier foreland, samples from 15 field‐collected plant species, 28 stomach and gut samples of seven cormorants, and 25 fecal pellets of salamanders. All samples stem from different projects run within the working group Applied and Trophic Ecology at the Institute of Ecology, University of Innsbruck.

In total, 185 samples (117 invertebrates, 53 vertebrates, and 15 plants) were DNA‐extracted with both CTAB and BioSprint^®^ 96 Blood and Tissue Kit protocols, resulting in 355 DNA extracts to be tested with both general and prey‐specific primers. The 15 plant DNA extracts were tested with general plant primers only. The original samples are stored at −80°C at the University of Innsbruck. Details on the different sample types, their collection, and origin are provided in Data S1 and S2. Species lists, individual DNA detections, prey detection postfeeding in feeding experiments with *Coccinella septempunctata,* breakdown of fish species detected in the stomach, fore‐, mid‐, and hindgut of the cormorants can be obtained from Data S2.

### 2.1 Lysis and DNA extraction

For the field‐collected predatory arthropods, the ladybird beetles from the feeding experiment, the insect omnivorous *Amara* sp. specimen, and the plant samples, the lysis was conducted as follows: Whole specimens and plant tissue, respectively, were put individually in 2‐ml reaction tubes adding 430 μl 1 × TES buffer (0.1 mol/L TRIS, 10 mmol/L EDTA, 2% SDS; pH 8), 10 μl proteinase K (20 mg/ml), and 5–8 glass beads (Ø 3 mm) each. After grinding in a Precellys^®^ 24 Tissue Homogenizer (Bertin Technologies, Montigny‐le‐Bretonneux, France) at 21,000 *g* for 2 × 60 s, they were incubated at 58°C for 24 hr. For plants and *Amara* sp., an additional 1 mg PVP (polyvinylpyrrolidone) was added to the lysis buffer to remove PCR‐inhibiting phenolic compounds stemming from plants. The vertebrate samples (stomach/gut content of cormorants, fecal pellets of salamanders) were treated similarly, without the grinding step, whereas for the cormorant samples, we raised the volume of 1 × TES buffer and proteinase K to 980 μl and 20 μl, respectively, due to their bigger initial volume. Per lysate, 200 μl each was used for CTAB and BioSprint DNA extraction.

The CTAB extraction was performed as follows: 60 μl of 5 mol/L NaCl and 25 μl of 10% CTAB (cetyltrimethylammonium bromide) solution were added to the 200 μl lysates and incubated for 10 min at 65°C. Next, 300 μl of chloroform:isoamylalcohol (24:1) was added, and the samples were allowed to rest for 10 min before they were centrifuged for 5 min at 18,000 *g*, and the aqueous layer transferred into fresh reaction tubes. After adding 100 μl of 5 mol/L NH_4_Ac, samples were placed on ice for at least 30 min and centrifuged for 20 min at 4°C 21,000 *g*, and the liquid phase was transferred into a fresh tube. DNA was precipitated with the same volume of isopropanol (approx. 350 μl) at −28°C overnight, and samples were centrifuged for 20 min at 4°C 21,000 *g*, washed with 300 μl 70% chilled ethanol, and again centrifuged at 4°C 21,000 *g* for 15 min. After removing the ethanol and air‐drying the pellet, DNA was finally resuspended in 200 μl 1 × TE (10 mmol/L TRIS, 1 mmol/L EDTA, pH 8.0) and stored at −28°C.

The automated DNA extraction was performed using the BioSprint^®^ 96 DNA Blood Kit on a BioSprint^®^ 96 extraction robotic platform (Qiagen). The kit combines silica‐based DNA purification with paramagnetic beads, where DNA binds to the silica surface of the paramagnetic particles in the presence of a chaotropic salt and is then washed repeatedly, making use of the same chemistry as the silica‐column‐based DNeasy Blood & Tissue Kit (Qiagen). We followed the manufacturer's instructions, with the exception that DNA was finally eluted in 200 μl 1 × TE buffer instead of buffer AE (10 mmol/L Tris–HCl, 0.5 mmol/L EDTA, pH 9.0) included in the kit. DNA extracts were stored at −28°C until PCR.

All DNA extractions were carried out in a separate pre‐PCR laboratory using a UVC‐equipped laminar flow hood. Two extraction negative controls were included in each batch of 30 samples to check for sample cross‐contamination. Likewise, within each PCR, at least one negative control (PCR water instead of template DNA) and one positive control (target DNA) were run to check for DNA carry‐over contamination and amplification success, respectively.

### 2.2 PCR, visualization, and statistical analysis

CTAB and BioSprint samples were initially tested in a PCR using general primers to check whether they contain amplifiable DNA and then with prey‐specific primers to specifically assess the detection of prey DNA in the samples (primer details and the respective PCR conditions are provided in Data S2). PCR products were visualized using QIAxcel^®^, an automated capillary electrophoresis system (Qiagen), with method AL320 on the QIAxcel Screening Kit, and the results were scored with BioCalculator Fast Analysis Software version 3.0 (Qiagen). Samples showing the expected fragment length with a signal above 0.05 and 0.1 relative fluorescent units were deemed positive, depending on the PCR assay.

To compare the performance of the different extraction methods, we used the PCR amplification success with both general and prey‐specific primers. Dietary samples are a mixture of only minute amounts of prey DNA together with high concentrations of predator DNA (King et al., 2008). Therefore, a quantification of prey DNA yield via devices such as Nanodrop^®^ or Qubit^®^ is impossible. Differences in DNA detectability between CTAB and BioSprint samples were statistically compared by chi‐square tests. All tests were carried out with SPSS 21 (IBM, Armonk, NY, USA).

### 2.3 Expenditure of time and money

Costs for DNA extraction per sample (excluding the lysis step) were estimated in terms of time and material expenses. Material affordability was calculated based on the list price for necessary supplies and reagents (as of January 2017). Start‐up costs for the BioSprint^®^96 as well as standard laboratory equipment such as centrifuges, thermo‐block, laminar flow, and extraction hood were excluded. Also the payment of the work was excluded as it is strongly dependent on the employment contract of the executing person.

## 3 RESULTS

### 3.1 Extraction efficacy via PCR amplification success

**Figure 1:**
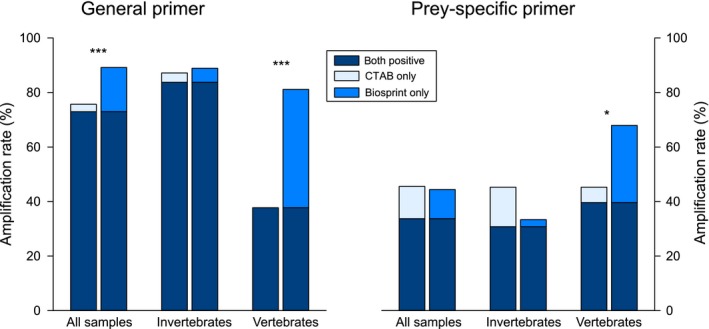
DNA amplification rates in invertebrate (*n* = 117) and vertebrate samples (*n* = 53) extracted with CTAB protocol and the BioSprint^®^ platform together with the DNA Blood Kit using general primers (left) and prey‐specific primers (right); for all samples taken together and separately for invertebrates and vertebrates, respectively. Amplification success for plant samples was 100% for both extraction methods (data not shown). *CTAB only* represents the share of samples that tested positive when they were CTAB‐extracted and negative when using BioSprint^®^ 96. For *BioSprint only,* this was exactly the other way round, that is, the share of samples that tested positive for BioSprint^®^ 96 and negative for CTAB. *Both positive* is the share of samples with successful PCR amplifications for both extraction methods. Asterisks indicate significant differences

For general primers, PCR amplification success was high across all samples (Figure 1). Here, the BioSprint extraction method generally turned out to be more effective in isolating amplifiable DNA than CTAB, as its performance was significantly higher for both groups together, vertebrates and invertebrates (χ^2^ = 10.9, *p* < .001). Among those samples which tested positive (i.e., successful PCR amplification) with only one of the two DNA extraction methods, the BioSprint extraction method had six times higher PCR amplification success than the CTAB extraction (Figure 1). This effect is mainly rooted in the large difference in amplification success for the vertebrate samples (stomach/gut content of cormorants and salamander feces) between BioSprint and CTAB (81.1% vs. 37.7%; χ^2^ = 20.7, *p* < .001). For the invertebrate samples, there was no such difference present (87.2% vs. 88.9% n.s.). All plant and ladybird beetle samples tested positive with both extraction methods using general primers (Table 1).

**Table 1:** Overview of the DNA detection success of the samples comprising different vertebrate, invertebrate, and plant taxa. (i) Invertebrates, (v) vertebrates; targeted genes: COI (mtDNA), 16S (mtDNA), CDH1 (nDNA), 18S (nDNA), and *trn*L (cpDNA). *CTAB only* (%) represents the share of samples that tested positive when they were CTAB‐extracted and negative when using BioSprint^®^ 96. For *BioSprint only* (%), this was exactly the other way round. *Both positive* (%) is the share of samples with successful PCR amplifications for both extraction methods and *Both negative* (%) is those which never delivered a PCR product

Consumer taxon	*n*	Target gene/Prey specificity	Fragment length (bp)	CTAB only (%)	BioSprint only (%)	Both positive (%)	Both negative (%)
General primes							
Carabidae (i)	25	COI	700	4.0	24.0	56.0	16.0
Spiders (i)	25	COI	700	8.0	0.0	80.0	12.0
*Coccinella septempunctata* (i)	52	COI	700	0.0	0.0	100.0	0.0
*Amara* sp. (i)	15	COI	700	6.7	0.0	80.0	13.3
*Phalacrocorax carbo sinensis* (v)	28	COI/CDH1	650/450	0.0	46.4	46.4	7.1
*Plethodon glutinosus/teyahalee* (v)	25	COI	700/350	0.0	40.0	28.0	32.0
Plants	15	*trn*L	120	0.0	0.0	100.0	0.0
Prey‐specific primers							
Carabidae (i)	25	18S (Collembola)	177	16.0	4.0	8.0	72.0
Spiders (i)	25	18S (Collembola)	177	16.0	4.0	16.0	64.0
*Coccinella septempunctata* (i)	52	COI (aphids)	231	5.8	1.9	46.2	46.2
*Amara* sp. (i)	15	*trn*L (plants)	120	40.0	0.0	40.0	20.0
*Phalacrocorax carbo sinensis* (v)	28	16S (fish)	172–383	7.1	21.4	46.4	25.0
*Plethodon glutinosus/teyahalee* (v)	25	18S (Collembola)	177	4.0	44.0	36.0	16.0

With the prey‐specific primers, prey DNA detection rates for all samples together were similar for both kinds of extraction methods: CTAB 45.6% and BioSprint extraction 44.4%, respectively (Figure 1). However, the situation changed when considering vertebrates and invertebrates separately: in vertebrate samples, the amplification success of prey DNA was significantly higher for BioSprint samples (73.6%) than for CTAB samples (47.2%, *p* < .005). Contrastingly, for the invertebrate samples, less BioSprint samples tested positive for prey DNA (33.3%) compared to the CTAB samples (45.3%, *p* = .061; Figure 1). Overall, there was no group of invertebrates where BioSprint samples had a better performance than CTAB samples, whereas for vertebrates the opposite was true (Table 1).

Among the invertebrates, there were no *Amara* sp. samples with amplifiable DNA exclusively for BioSprint (i.e., they either were positive exclusively for CTAB or with both extraction methods), neither with the general nor with the prey‐specific primers. Contrastingly, among the vertebrates, no cormorant stomach/gut content samples tested positive exclusively for the CTAB extraction.

### 3.2 Expenditure of time and money

Material costs per sample were one‐third cheaper for CTAB DNA extraction than for the BioSprint^®^ 96 extraction platform using the BioSprint^®^ DNA Blood and Tissue Kit (Table 2). Yet, the time expenditure for CTAB samples was over eight times higher than for BioSprint samples. The CTAB extraction implies two transfers per sample of parts of the lysate from one reaction tube to a fresh one. Moreover, reaction tubes need to be opened and closed six times during the procedure in order to add reagents. Contrastingly, the BioSprint samples are only opened once, that is, when the lysate is transferred into the 96‐well plate going into the extraction device. This could lower the risk of carry‐over contamination.

**Table 2:** Overview of the expenditure of time and money per sample (excluding the lysis step) comparing DNA extraction with a CTAB protocol and the BioSprint^®^ 96 extraction platform using the BioSprint^®^ Blood and Tissue Kit (Qiagen, Hilden, Germany). *Time* indicates hands‐on time per sample; *Costs* includes all disposals and reagents needed in € (list price Jan 2017); *Times Opening Tubes* defines how often it is necessary to open and close a reaction tube during the extraction procedure. Start‐up costs for BioSprint^®^ extraction platform as well as standard laboratory equipment were excluded

Method	Time (min)	Costs (€)	Times opening tubes	Risk of cross‐contamination
BioSprint	1.8	1.3	1	Low
CTAB	15	0.89	6	High

## 4 DISCUSSION

We compared the performance of a high‐throughput DNA extraction procedure, with the example of the BioSprint^®^ 96 extraction platform in combination with the commercial BioSprint^®^ 96 DNA Blood and Tissue Kit, and a classical, well‐proven CTAB‐based protocol for the detection of prey DNA in dietary samples from whole‐body invertebrate samples, vertebrate stomach/gut contents, and feces. Using general primers, the two extraction methods resulted in similar rates of amplifiable total DNA, meaning that the PCR amplification success for total DNA was over 90% in both cases. Here, the performance of the BioSprint samples was higher for both vertebrates and invertebrates. This indicates that automated magnetic separators are more effective in extracting total DNA than the CTAB‐based protocol. Moreover, automatic DNA extraction procedures have the huge advantage that they generally significantly decrease time and costs. Ivanova, DeWaard, and Hebert (2006), Ivanova, Fazekas, and Hebert (2008), for example, optimized a semiautomated DNA extraction method for animal tissue and plants using glass fiber 96‐well plates (PALL Inc.) in combination with a Biomek^®^ FX liquid handling station (Beckman Coulter). The performance of this method was comparable to single‐tube commercial DNA isolation kits. However, Ivanova's approach still implies numerous steps of handling with plates and chemicals during the extraction process, whereas devices such as BioSprint^®^ are designed for fully automated DNA extraction once loaded with lysate and buffers. Because of this, the risk of contamination is significantly reduced, allowing for standardized DNA extraction procedures. This can be seen, for example, in high‐throughput protocols using paramagnetic separation techniques in combination with automated DNA extraction systems for detecting enteric livestock diseases in feces (Chen et al., 2014; Plain et al., 2014). Additional benefits of the plate‐based DNA extraction of automated platforms in comparison with individual tube‐based extractions are the overall reduction in plastic waste and the usually smaller reagent volumes required (Schiebelhut, Abboud, Gómez Daglio, Swift, & Dawson, 2016).

Automated DNA extraction has not yet been evaluated for dietary samples. The present results indicate significant differences between the two extraction methods when using prey‐specific primers for vertebrates and invertebrates, with higher prey DNA detection rates for BioSprint samples in vertebrates. The results are considered to be representative for any of the platforms using this well‐proven principle that are offered by various companies. The Kingfisher^®^ 96 Purification System (Thermo Fisher), for example, is nearly identical to the platform we have used. Prey DNA detection success in the cormorant BioSprint samples was similar to the dietary studies on penguins (Jarman et al., 2013), where a “Maxwell 16” DNA extraction robot (Promega) was used, also working with paramagnetic beads. The contrasting higher recovery rate of prey DNA in CTAB‐ compared to BioSprint samples of invertebrates may be rooted in the fact that the former extraction protocol represents a constantly improved procedure of working steps, where most of the optimization was performed in regard to the detection of prey DNA in whole‐body extracts of invertebrates (Juen & Traugott, 2005; Oehm et al., 2011; Raso et al., 2014; Wallinger et al., 2013). We kept on continuously adapting and optimizing these CTAB protocols depending on our needs for different predator and “prey” species (invertebrates, vertebrates, plant tissue, seeds). Over the last 10 years, we constantly tested them against commercially available DNA extraction kits of various suppliers. Independent of which other kit/method we used in the past, the CTAB protocol was the best. For the extraction via BioSprint^®^ 96, however, just a few amendments of the manufacturers' protocol have been performed so far, mainly regarding the lysis step. Besides, the differences in prey DNA detection between CTAB and BioSprint samples may be caused by the different nature of sample types in vertebrates and invertebrates: Contrastingly to the vertebrate samples, where only stomach/gut content (cormorants) and feces (salamanders) were used, invertebrates were extracted as a whole including the prey in their guts. This leads to high concentrations of consumer DNA together with minute amounts of prey DNA (King et al., 2008). In vertebrate feces, a considerable fraction of DNA can originate from cells of the intestinal mucosa of the defecating animal, too (Albaugh et al. 1992; Deagle et al. 2006). However, the share of consumer DNA is far below the one in whole‐body invertebrate extracts. The weaker performance of BioSprint^®^ 96 for prey DNA detection in invertebrates might be attributable to the fact that the silica surface of the paramagnetic beads is fully occupied by consumer DNA, so that there is no more binding capacity left for prey DNA. A potential solution here would be the use of invertebrate regurgitates (Waldner & Traugott, 2012; Wallinger et al., 2015) or feces (Sint et al., 2015) instead of whole‐body extracts. Alternatively, the weaker performance of BioSprint samples could be explained by a weaker affinity of the silica surface to short DNA fragments (BioSprint^®^ Handbook, Qiagen) resulting from preceding digestion in the consumer. Altogether, according to the present results, the CTAB extraction protocol seems to be more efficient when targeting shorter amplicons of invertebrates including higher concentrations of nontarget DNA, whereas the particular strength of automated magnetic separators such as BioSprint^®^ 96 involves the binding of longer DNA fragments. This is also suggested by the screening of the cormorant samples with the prey‐specific FishTax assay: Here, the fragment length of detected amplicons exclusively after CTAB extractions (i.e., the corresponding BioSprint samples were negative; *n* = 5) tended to be shorter than the ones exclusively after the BioSprint^®^ 96 extraction (*n* = 9; details are provided in Data S2. Among the vertebrates, BioSprint samples had a much higher performance in prey DNA detection than the CTAB samples. A possible explanation for the weaker performance of the CTAB samples could be the presence of inhibitory substances in the (semi)digested material (Zarzoso‐Lacoste et al., 2013). A major advantage when using automated separators is that the paramagnetic particles together with the bound DNA are transferred between different tubes (BioSprint^®^ 96 User Manual). Hence, DNA is specifically bound from the lysate, whereas when CTAB extracting, all unwanted substances apart from DNA are removed. Consequently, DNA molecules that nonspecifically stick at the inner surface of the reaction tube are excluded when using automated separators. In this manner, substances other than nucleic acids which might potentially inhibit PCR are removed more reliably than with the CTAB protocol. The present results are in accordance with previous studies comparing DNA extraction methods of dietary samples in vertebrates and invertebrates (Oehm et al., 2011; Simonelli et al., 2009), suggesting that the detection and identification of feeding relationships are susceptible to experimental factors associated with prey DNA isolation procedures. Sample type and length of the targeted fragment seem to impact PCR amplification success of the different extraction methods. Therefore, for achieving reliable and robust results to correctly interpret the complex trophic interplay of the species involved, consideration should be taken when choosing an extraction method appropriate for the species of interest and changes of extraction methods during studies should be avoided.

One of the biggest advantages of employing robotic DNA extraction platforms is that they save time as they speed up the analysis and reduce analytical error. Compared to classical DNA extraction approaches, their use in combination with commercial kits allows to run up to 96 samples at once with minimal hands‐on time (Carter et al., 2010). Albeit such automated systems are not cheap, it is paying off in a rather short time at a corresponding use of capacities and eventually a joint usage among different labs. Furthermore, automated platforms reduce the risk for cross‐contamination due to the few manual handling steps compared to CTAB‐ or column‐based silica extraction protocols. Among these platforms, BioSprint^®^ 96 or Kingfisher^®^ 96 offer the advantage of being “open systems” allowing tailoring of one's own methods. Although predefined application protocols are available, the results of the present study indicate the relevance of an option for customizing existing protocols or creating entirely new ones to meeting specific requirements. For example, different mixing speeds turned out to have a strong effect on the DNA purification (Suomalainen et al., 2010).

In conclusion, paramagnetic particle‐based DNA purification systems, employing an automated platform such as BioSprint^®^ 96, achieve high rates of amplification success for total DNA form a variety of samples. Moreover, the method proved to be rapid, efficient, and reliable for DNA extraction also in different types of dietary samples. The system tested here is highly recommendable for large‐scale dietary studies. The drastically reduced time effort and the low risk of cross‐contamination play a critical role in high‐throughput analysis. The system has a number of advantages regarding laboratory use: Parallelization allows for simultaneous handling of different targets and increased throughput. Moreover, the risk of cross‐contamination is drastically reduced. Although there is still scope for improvement, the present results demonstrate that paramagnetic bead‐based DNA extraction in combination with the use of automated extraction platforms is a promising tool for investigating trophic interactions comprehensively and is highly recommendable for high‐throughput analysis.

## AUTHOR CONTRIBUTIONS

DS, KS, BT, CW, and MT conceived and designed research. DS conducted feeding experiments with *Coccinella septempunctata*. KS, CW, BT, JO, and AJ collected dietary samples of invertebrates (KS, CW) and vertebrates (BT, JO, AJ) and conducted the molecular work. DS and CW analyzed the data. CW and MT wrote the manuscript. All authors read and contributed to the final version of the manuscript.

## DATA ACCESSIBILITY

The data of field experiment and molecular analysis will be uploaded to DRYAD upon publication.

## CONFLICT OF INTEREST

None declared.
