# Supplementary material for: Evaluation of an automated protocol for efficient and reliable DNA extraction of dietary samples
Source: Ecol Evol. 2017 Jul 7;7(16):6382–9. doi: 10.1002/ece3.3197 (PMC5574753; doi:10.1002/ece3.3197)
Supplement: Supplementary file 2 [file ECE3-7-6382-s002.docx]

***Data S2 Sample collection, primer details and PCR conditions***

***Detection of total DNA with general primers***

For the field-collected predatory arthropods and the salamander faeces, the general primers LCO1490 (GGTCAACAAATCATAAAGATATTGG) and HCO2198 (TAAACTTCAGGGTGACCAAAAAATCA), targeting the cytochrome *c* oxidase subunit one (COI) gene (Folmer *et al.* 1994) were employed. The salamander samples were additionally tested with the primer combination C1-J-1859 (GGAACXGGATGAACTGTTTACCCXCC (Simon *et al.* 1994) and HCO2198 (Folmer *et al.* 1994). The 15-µl PCRs contained 1.5 µl template DNA, 0.2 mM dNTPs, 0.5 µM of each primer, 1×OneTaq Buffer with MgCl_2_, 0.375 U OneTaq (New England Biolabs, Frankfurt/Main, Germany), 1 mM MgCl_2_ (to increase the final concentration to 2.8 mM), 7.5 µg BSA (bovine serum albumin), and molecular grade water to adjust the volume. The thermocycling programme was 94 °C for 30 s, 35 cycles of 94 °C for 15 s, 50 °C for 60 s, 68 °C for 60 s, and a final elongation at 68 °C for 5 min.

For the ladybird beetles from the feeding experiment and the *Amara* sp. specimens from the glacier foreland, the same general COI primers as described above (LCO1490 and HCO2198 (Folmer *et al.* 1994) were employed in 10µL reactions, containing 1.5µl template DNA, with 1.5 µl template DNA, 0.2 mM dNTPs, 0.5 µM of each primer, 1×OneTaq Buffer with MgCl_2_, 0.375 U OneTaq, 1.2 mM MgCl_2_ (to increase the final concentration to 3 mM), and 5 µg BSA. The cycling conditions were 94 °C for 120 s, 35 cycles of 94 °C for 20 s, 50 °C for 30 s, 68 °C for 60 s, and a final elongation at 68 °C for 3 min. The plant samples were tested using the PCR assay described in Wallinger *et al.* (2013). It employs the general plant primer *c* A49325 (CGAAATCGGTAGACGCTACG), accessing the *trn*L (UAA) exon (Taberlet *et al.* 1991) and *trn*L110R (GATTTGGCTCAGGATTGCCC), located in the *trn*L (UAA) intron (Borsch *et al.* 2003). For the cormorants samples, we used general primers targeting the COI, C1-J-1859 and C1-N-2329 (ACTGTAAATATATGATGAGCTCA; (Simon *et al.* 1994), as well as 2550F (GTTACTGATTCGTCTACGAGA) and 2718R (ATTGAAATGATCCAGTGCTTG), targeting the chromodomain-helicase-DNA-binding protein 1 (CHD1) gene (Fridolfsson & Ellegren 1999). The PCRs were run in 10 µl reactions with 1.5 µl template DNA, 0.2 mM dNTPs, 0.5 µM of each primer, 1×OneTaq Buffer with MgCl_2_, 0.375 U OneTaq, 1.2 mM MgCl_2_ (to increase the final concentration to 3 mM), and 5 µg BSA with a thermocycling program starting with 94 °C for 2 min, followed by 35 cycles of 94 °C for 20 s, 50 °C for 30 s, 68 °C for 60 s, and a final elongation at 68 °C for 3 min. The PCR conditions for prey detection are described below for the respective sample type.

***Field-collected predatory arthropods***

Twenty five carabid beetles and 25 spiders (14 Lycosidae, 9 Linyphiidae, 1 Tetragnathidae, 1 Theridiidae) were collected in spring 2011/12 in cereal fields in Sweden (counties of Uppsala and Scania) and frozen at -28° C upon collection (detailed description of field sampling see in (Staudacher *et al.* 2016). Their DNA extracts were tested for prey DNA with collembolan-specific primers, since they are frequently consumed by predators from these families (Kuusk & Ekbom 2012, Raso et al. 2014, Davey et al. 2011). The primers Col4F (GCTACAGCCTGAACAWTWG) and Col5R (TCTTGGCAAATGCTTTCGCAGTA) target the 18S rDNA region (Kuusk & AgustÍ 2008). The PCR ran in 10 µl reactions containing 1.5 µl template DNA, 1×Multiplex PCR Master Mix (Qiagen), 0.4 µM of each primer, and a thermocycling program with 15 min at 94 °C, 35 cycles of 94 °C for 30 s, 61 °C for 90 s, 72 °C for 90 s, and a final elongation at 72 °C for 10 min.

***Feeding experiment with ladybird beetles***

Adult ladybird beetles (*Coccinella septempunctata*) and English grain aphids (*Sitobion avenae*) were obtained from Katz Biotech AG (Baruth, Germany). The beetles were kept individually in transparent 20 ml plastic tubes containing a piece of moistened tissue, in a climate cabinet at 20 °C. Tubes were ventilated and the tissues replaced every second day. Prior to the feeding experiments, beetles were starved for 16 – 24 hrs before consuming 1 aphid each. Batches of 13 individuals were frozen at -28° C at 0, 8, 16, and 24 h post-feeding. Prey detection was performed in 10 µl reactions, containing 2.5 µl template DNA, 1×Multiplex PCR Master Mix (Qiagen), 0.2 µM of each primer (Sit-ave-S103 (ACATTTAGCAGGAATCTCATCA), and Sit-ave-A103 (TCTCCTCCTCCTGCTGGA) (Traugott *et al.* 2012) with 95 °C for 15 min, 35 cycles of 94 °C for 30 s, 62 °C for 3 min, 72 °C for 60 s, and a final elongation at 72 °C for 10 min.

For both extraction protocols, identical DNA detection rates were achieved with the ladybird beetle samples tested at 0 h (100%) and 24 h (15%) post-feeding (Fig. 1). At 24 h post-feeding two samples amplified only with one of the two extraction methods. At 8 h and 16 h post feeding, each one sample tested positive when extracted with CTAB but no aphid DNA could be amplified from the BioSprint-sample, so that overall the detection success was marginally higher after CTAB extraction.

**Figure 1** Detectability of prey DNA in ladybird beetles (*Coccinella septempunctata*), fed with aphids, at four different time points ranging from 0 to 24 h post-feeding. Thirteen individuals per time point post-feeding were tested with prey species-specific primers. Detection rates are provided for the different time points as circles for the CTAB- and as triangles for the BioSprint-samples.

***Field-collected omnivorous beetles and plants***

A total of 15 field-collected omnivorous carabid beetles belonging to the genus *Amara* were caught in a Central Alpine glacier valley located near Obergurgl (Tyrol, Austria) at approx. 2400 m a.s.l. (detailed description of field sampling in Raso (2013). The collected beetles were kept individually in 1.5 ml reaction tubes at -80 °C until DNA extraction. Plant DNA detection in the omnivores’ guts was done using the PCR assay described in Wallinger et al. (2013). Individuals of 15 different plant species representing 10 families (for details see supplementary S1) were collected in arable grasslands around Innsbruck in summer 2013 and kept at -28 °C until DNA extraction.

***Stomach/gut content of cormorants***

Seven Great Cormorants (*Phalacrocorax carbo*) shot near the river Lech were obtained from the fish farm of the Bavarian Environment Agency (LfU) in Wielenbach (Bavaria, Germany). Upon dissection, the digestive tract of each bird was divided into four parts, the stomach, the foregut, the mid-gut, and the hind-gut. Scraping samples of the undigested fish and of the mucosa were taken from the stomach and put into a 1.5 ml reaction tubes. The gut content of the three remaining gut parts of the digestive tract was squeezed separately into Petri dishes, mixed and approximately 0.5 ml were transferred into 1.5 ml reaction tubes (for details of the bird collection and sampling see Oehm *et al.* (2016). Prey detection was performed with fish-specific primers using the FishTax multiplex PCR assay as described in Thalinger *et al.* (2016).

**Table 1** Results of 7 cormorant digestive tracts (4 samples per cormorant; A-D from stomach to fore- and hindgut) tested for the presence of fish DNA with the FishTax multiplex PCR assay (Thalinger *et al.* 2016). ‘CTAB’ – fragment only amplified from CTAB-sample, ‘BioSp’ – fragment only amplified from BioSprint-sample, ‘both’ – fragment amplified from both DNA extracts. Targets included in FishTax: *Acipenser ruthenus* (A.rut), Siluriformes (Silu), *Anguilla anguilla* (A.ang), Salmoniformes (Salm), *Lota lota* (L.lot), *Esox lucius* (E.luc), Cypriniformes (Cypr), Percomorphaceae (Perc), Petromyzontidae (Petr).

| **Sample** | **A.rut**  (109 bp) | **Silu**  (149 bp) | **A.ang**  (172 bp) | **Salm**  (195 bp) | **L.lot**  (237 bp) | **E.luc**  (265 bp) | **Cypr**  (290 bp) | **Perc**  (383 bp) | **Petr**  (405 bp) |
| --- | --- | --- | --- | --- | --- | --- | --- | --- | --- |
| K25-A | 0 | 0 | 0 | 0 | 0 | both | CTAB | 0 | 0 |
| K25-B | 0 | 0 | 0 | 0 | 0 | BioSp | 0 | 0 | 0 |
| K25-C | 0 | 0 | 0 | 0 | 0 | BioSp | 0 | 0 | 0 |
| K25-D | 0 | 0 | 0 | 0 | 0 | BioSp | 0 | 0 | 0 |
| K26-A | 0 | 0 | 0 | 0 | CTAB | 0 | 0 | 0 | 0 |
| K26-B | 0 | 0 | 0 | 0 | 0 | 0 | 0 | 0 | 0 |
| K26-C | 0 | 0 | 0 | 0 | 0 | 0 | 0 | 0 | 0 |
| K26-D | 0 | 0 | 0 | 0 | BioSp | 0 | 0 | 0 | 0 |
| K31-A | 0 | 0 | 0 | 0 | 0 | 0 | 0 | 0 | 0 |
| K31-B | 0 | 0 | 0 | 0 | 0 | 0 | 0 | 0 | 0 |
| K31-C | 0 | 0 | 0 | 0 | 0 | 0 | 0 | 0 | 0 |
| K31-D | 0 | 0 | 0 | 0 | 0 | 0 | 0 | 0 | 0 |
| K42-A | 0 | 0 | 0 | both | 0 | both | 0 | 0 | 0 |
| K42-B | 0 | 0 | 0 | both | 0 | 0 | 0 | 0 | 0 |
| K42-C | 0 | 0 | 0 | both | 0 | BioSp | 0 | 0 | 0 |
| K42-D | 0 | 0 | 0 | CTAB | 0 | 0 | 0 | 0 | 0 |
| K103-A | 0 | 0 | 0 | 0 | 0 | 0 | both | both | 0 |
| K103-B | 0 | 0 | 0 | 0 | 0 | 0 | both | 0 | 0 |
| K103-C | 0 | 0 | 0 | 0 | 0 | 0 | both | 0 | 0 |
| K103-D | 0 | 0 | 0 | 0 | 0 | 0 | both | 0 | 0 |
| K106-A | 0 | 0 | 0 | 0 | 0 | both | 0 | BioSp | 0 |
| K106-B | 0 | 0 | 0 | 0 | 0 | both | 0 | 0 | 0 |
| K106-C | 0 | 0 | 0 | 0 | 0 | both | 0 | 0 | 0 |
| K106-D | 0 | 0 | 0 | 0 | 0 | both | 0 | 0 | 0 |
| K113-A | 0 | 0 | CTAB | 0 | 0 | CTAB | both | both | 0 |
| K113-B | 0 | 0 | 0 | 0 | 0 | 0 | 0 | 0 | 0 |
| K113-C | 0 | 0 | 0 | 0 | 0 | 0 | 0 | BioSp | 0 |
| K113-D | 0 | 0 | 0 | 0 | 0 | 0 | BioSp | BioSp | 0 |

***Faecal pellets of salamanders***

In the summer 2012, salamanders of the species *Plethodon glutinosus* c.f. *teyahalee* were collected in the Great Smoky Mountains National Park, located at the border between Tennessee and North Carolina (USA) (for a detailed description of the sampling see (Straube 2013). Animals were put in petri dishes on a piece of moistened filter paper and brought to a laboratory where they were kept until defecation at 20 °C constant in the dark. After defecation, faecal pellets were individually frozen at -28 °C and salamanders were released in their original habitat. The 25 obtained faecal samples were tested for prey DNA using the collembolan-specific primers and PCR conditions described above for the field-collected generalist predatory arthropods.

***References***

Borsch T, Hilu K, Quandt D*, et al.* (2003) Noncoding plastid trnT-trnF sequences reveal a well resolved phylogeny of basal angiosperms. *Journal of Evolutionary Biology* **16**, 558-576.

Folmer O, Black M, Hoeh W, Lutz R, Vrijenhoek R (1994) DNA primers for amplification of mitochondrial cytochrome c oxidase subunit I from diverse metazoan invertebrates. *Molecular Marine Biology and Biotechnology* **3**, 294–299.

Fridolfsson A, Ellegren H (1999) A simple and universal method for molecular sexing of non-ratite birds. *Journal of Avian Biology* **30**, 116-121.

Kuusk A, AgustÍ N (2008) Group-specific primers for DNA-based detection of springtails (Hexapoda: Collembola) within predator gut contents. *Molecular Ecology Resources* **8**, 678-681.

Oehm J, Thalinger B, Mayr H, Traugott M (2016) Maximising dietary information from avian cadavers. *IBIS*, in press.

Raso L (2013) *Pioneer predator communities in glacier forelands* monography, University of Innsbruck.

Simon C, Frati F, Beckenbach A*, et al.* (1994) Evolution, weighting, and phylogenetic utility of mitochondrial gene sequences and a compilation of conserved polymerase chain reaction primers. *Ann. Entomol. Society of America* **87**, 651-701.

Staudacher K, Jonsson M, Traugott M (2016) Diagnostic PCR assays to unravel food web interactions in cereal crops with focus on biological control of aphids. *Journal of Pest Science* **89**, 281-293.

Straube D (2013) *Development and application of molecular techniques to assess feeding interactions in a forest invaded by the Asian earthworm Amynthas agrestis*, University of Innsbruck.

Taberlet P, Gielly L, Pautou G, Bouvet J (1991) Universal primers for amplification of three non-coding regions of chloroplast DNA. *Plant Molecular Biology* **17**, 1105-1109.

Thalinger B, Oehm J, Mayr H*, et al.* (2016) Molecular prey identification in Central European piscivores. *Molecular Ecology Resources*, n/a-n/a.

Traugott M, Bell J, Raso L, Sint D, Symondson W (2012) Generalist predators disrupt parasitoid aphid control by direct and coincidental intraguild predation. *Bulletin of Entomological Research* **102**, 239-247.

Wallinger C, Staudacher K, Schallhart N*, et al.* (2013) The effect of plant identity and the level of plant decay on molecular gut content analysis in a herbivorous soil insect. *Molecular Ecology Resources* **13**, 75–83.
